# Re-Expanding Opportunities for American Descendants of Slavery to Build a More Inclusive and Diverse Global Health Workforce

**DOI:** 10.4269/ajtmh.21-0720b

**Published:** 2021-08-09

**Authors:** Tishina Okegbe

**Affiliations:** Social Solutions International, Inc.Rockville, Maryland E-mail: tokegbe@socialsolutions.biz

Dear Sir,

I would like to thank Dr. Richards for his thoughtful response to my perspectives piece, and the opportunity from *American Society of Tropical Medicine and Hygiene* (*AJTMH*) to respond.^1^ I am always happy to engage in dialogue to keep the topic of under representation of American descendants of slavery (ADOS) in the global health field at the forefront.

As I acknowledge in my perspectives piece, the term ADOS is relatively new and still controversial—however, it captures a nuance that has been missed by many other terms used to describe the subpopulation of melanated people with generational roots in the United States, including Black and African American. On this point, I imagine that Dr. Richards would agree. Further, I know that individuals may have varying levels of comfort using the ADOS label. One of the central goals of the movement to highlight the ADOS concept is to advocate for reparations for the system of slavery and to raise awareness about the injustices that ADOS continue to face in the United States. In short, the ADOS movement seeks to highlight the struggles descendants of enslaved people have faced since emancipation was declared in 1865, rather than to “subliminally negate” those sufferings.^2^

Second, I appreciate Dr. Richards’ reading of my article with his “physician’s brain turned on.” However, as a nonclinical professional, I view “acute” in layperson’s terms. According to the Oxford Languages Dictionary, acute is an adjective that describes *a bad, difficult, or unwelcome situation or phenomenon* that is *present or experienced to a severe or intense degree*.^3^ The dearth of ADOS representation in the global health field is most certainly severe, and as Dr. Richards pointed out, longstanding.

Third, I value Dr. Richards’ additional historical example and agree that ADOS have accomplished great feats on the global stage, and that these feats should be celebrated. However, I would caution that the scope of my article is narrowly focused on ADOS representation within the global health field and would welcome further examples that highlight this specific demographic.

Finally, I wholeheartedly agree that capturing ADOS representation in the global health field is required to adequately measure progress and inclusion over time, as I note in my article; however, I also acknowledge that we are far from making this a reality. The *Benchmarking Race, Inclusion & Diversity in Global Engagement* survey was published in June and sought to “provide an industry-wide benchmark of the development and humanitarian industry in the US.”^4^ Of note, the survey found that one in four organizations did not capture any race data, and therefore had no idea of the racial/ethnic composition of its workforce. I am hopeful that, as organizations become more aware of the importance of collecting this type of basic demographic data, the possibility of establishing a monitoring database may not be so farfetched.

In closing, I am heartened by the responses to my article and happy it has sparked conversation. As a daughter of an immigrant parent and an ADOS parent, these musings are very real to me, and I long to see a future where ADOS are well represented and thriving in global health careers.
